# The magnitude of teenage pregnancy and its associated factors among teenagers in Dodoma Tanzania: a community-based analytical cross-sectional study

**DOI:** 10.1186/s12978-022-01554-z

**Published:** 2023-02-03

**Authors:** Fabiola V. Moshi, Olympia Tilisho

**Affiliations:** 1 Department of Nursing Management and Education, School of Nursing and Public Health, The University of Dodoma, P.O. Box 259, Dodoma, Tanzania; 2 Department of Clinical Nursing, School of Nursing and Public Health, The University of Dodoma, P.O. Box 259, Dodoma, Tanzania

**Keywords:** Teens, Pregnancy, Adolescence, Factors, Dodoma, Tanzania

## Abstract

**Background:**

Teenage pregnancies cause serious health, social and economic consequences including death among adolescent girls worldwide. It is estimated that in 2019 about 55% of unintended pregnancies among adolescent girls aged 15–19 years ended up in abortions, which are often unsafe in developing countries. Little was known about the magnitude of teenage pregnancy and its associated factors in Dodoma Tanzania. Therefore, the study aimed at establishing the magnitude and factors associated with teenage pregnancies among adolescents in Dodoma Region Tanzania.

**Method:**

It was a community-based analytical cross-sectional study that included 539 adolescent girls aged 15 to 19 years old. A multistage sampling technique was used to recruit study participants. An interviewer-administered structured questionnaire was used to collect data. Data were analyzed by using SPSS v23. Descriptive statistics were used to describe the distribution of the entire study variable while the inferential statistics helped to establish factors associated with teenage pregnancy among adolescent girls and the level of significance was set at two sides of less than 0.05.

**Results:**

The magnitude of teenage pregnancy in Dodoma was 29%. After controlling for possible confounders, factors associated with teenage pregnancy were; urban residence [AOR: 3.02, 95%CI: (1.60–5.68), p = 0.001], low mothers’ education status [AOR: 3.46, 95%CI: (1.47–8.11), p = 0.004]; Poor knowledge on sexual and reproductive health [AOR: 2.09, 95%CI: (1.2–3.63), p = 0.009]; Low family economic status [AOR: 3.83, 95%CI: (1.77–8.30), p = 0.001]; Peer group [AOR: 2.92, 95%CI: (1.33–6.40), p = 0.007]; Early marriage [AOR: 2.58, 95%CI: (1.57–4.26), p < 0.0001]; and Sexual abuse [AOR: 13.56, 95%CI: (7.98–23.06), p < 0.0001].

**Conclusion:**

This study found a high magnitude of teenage pregnancy among youth in Dodoma. Teenagers who were more likely to teenage pregnancy were those with limited knowledge about sexual and reproductive health living in urban, from families with low economic status, their mothers had a low level of education, from a culture that encourage early marriages, with the influence of peer and who experienced sexual abuse. An innovative intervention study to come up with a cost-effective strategy to address the challenge of teenage pregnancy in Dodoma is highly recommended.

## Plain English summary

The study is reporting the status of teenage pregnancy among adolescents aged 15–19 years in the Dodoma Region. Dodoma Region is the capital city of Tanzania and it is among the fastest-growing city of Tanzania. The region has the highest population of youth in Tanzania. In addition, the study is reporting on factors associated with teenage pregnancy. A range of factors that have been reported by previous studies was assessed in this study to test whether they are significant factors in this population. These factors were sociodemographic factors (age, sex, marital status, education, occupation), social factors (Peer pressure, sexual abuse), familial factors (Household socio-economic status, domestic violence, marital status,), behavioral factors (like age at first sexual intercourse, early marriage, and contraceptive use, perception on teenage pregnancy, family income, peer pressure, and casual sex) and knowledge on sexual and reproductive health. The findings from this study are key to the development of a focused intervention study that will work out the strategies to be used to solve the problem of teenage pregnancies in Dodoma Region Tanzania.

## Background information

Teenage pregnancy has become a global health problem in recent years. According to WHO globally nearly 16 million teenagers aged 15–19 years old and two million girls under the age of 15 years old give birth every year [1]. More than 90% of these births occur in low and middle-income countries [2]. In 2018, the prevalence of adolescent pregnancy in Africa was reported to be 18.8% and 19.3% in the Sub-Saharan African region. The East Africa region had the highest prevalence of teenage pregnancy at 21.5% [3].

Tanzania reported an increase in teenage childbearing from 23 to 27% in 2010 and 2016, respectively [4] with an increase in maternal mortality ratio from 446 [5] to 556 [6] maternal deaths per 100,000 live births; a high rate being among adolescent mothers(NBS, 2016). A total of 32% of teenagers who have begun childbearing are rural women (19%). Studies have shown that the worse affected are teenagers aged 15–19 and nearly 60% of risky abortion in Africa is among young girls [7].

The percentage of teenagers who have had a child or are pregnant is 27% in Tanzania Mainland, more than three times higher than in Zanzibar 8% [8]. There exist variations in teenage pregnancy in Tanzania, with Dodoma region being among the leading regions with teenage pregnancy ranging between 34 and 45% [4].

Teenage pregnancy is associated with serious social concerns such as poor education, hazardous behaviors that lead to underprivileged health issues, child welfare, and poverty [9]. Teenage pregnancies are of concern because they have negative demographic, socio-cultural, and socioeconomic consequences for parents [10]. Teenage pregnancies have major health consequences that leading cause of death among adolescent girls aged between 15 to 19 years worldwide [11]. An estimated 50% of pregnancies among teenage women in developing countries are unintended [12]. Most of these adolescent pregnancies end up in abortions; about five million abortions are estimated to occur among adolescent girls every year and about 70% are insecure, adding to maternal mortality, morbidity, and lasting health problems [13]. The study conducted in Tanzania in 2011 by UNICEF reported that most unsafe abortion is done by adolescent women with an estimated 30% of unsafe abortions that occurred to young women aged between 15 and 19 years [14].

Teenagers who are pregnant face risks of pregnancy and childbirth-related complications such as pre-eclampsia, infections, and puerperal endometritis; also increased danger of low birth weight, preterm delivery, and severe neonatal conditions [15]. Knowledge of sexual and reproductive health remains a major concern worldwide, its burden is very high in LMICs with a high rate of early pregnancy [16]. In addition to that, adolescent pregnancy is related to an increased danger of death, disability, and infectious diseases in early motherhood [12].

The young woman begins to be sexually active without having adequate information about pregnancy and the risks of sexually transmitted infections [17]. However, different measures have been taken by the government through the Ministry of health, and support from partners for health including WHO has been addressing education on teenage pregnancy, some teaching has been provided at schools and found in school syllabuses; despite the major actions taken by to prevent early motherhood, still, there is a rapid increase of teenage pregnancy, especially in SSA including Tanzania [18].

Inadequate access to reproductive health services among teenagers in low-income countries including Tanzania is cited as one of the predictors of teenage pregnancies [19]. Access to reproductive health services is a right for everybody regardless of gender and age. Therefore, it is the responsibility of the country to ensure the accessibility of reproductive health services to all individuals. This is because, if a teenager becomes pregnant, all of her expectations diminish.

Literature has cited behavioral factors such as having multiple sexual partners, frequent sex, and irregular contraceptive use as factors that increase the likelihood of teenage pregnancy among teenagers [2, 3, 20]. Other factors which are reported to increase the likelihood of teenage pregnancy were early marriage, peer pressure, sexual abuse, and lack of control over sex [3, 20]

In Tanzania, early motherhood is a growing burden, yet little is empirically stated about the major predictors contributing to early motherhood, neither the magnitude nor the determinants for specific areas of the country like Dodoma Region were established in the literature**.** Failure to identify the predictors of teenage pregnancy increases the burdens on public health facilities to prevent and reduce the risk of complications due to pregnancy and childbirth among adolescents.

## Methods

### Study design

The study was a community-based analytical cross-sectional study. The design was used to identify the magnitude and predictors of teenage pregnancy in the Dodoma Region, Tanzania.

### Study settings

This study was conducted at a community level in the Dodoma region situated in the central zone of Tanzania with seven districts. Dodoma is the official capital city of Tanzania. It is located 480 km west of the Indian Ocean in the center of the country. It covers an area of 2669 sqm3. The population of the Dodoma region has increased tremendously due to the migration of citizens from the upcountry.

### Study population

The study population was adolescent girls aged 13–19 years residing in the Dodoma region.

### Eligibility criteria

#### Inclusion criteria

Adolescent girls aged 13–19 years residing in Dodoma Region during the period of study and whose parents/caretakers signed the consent form for voluntary participation in this study.

#### Exclusion criteria

Adolescent girls aged 13–19 years who were sick during the data collection, who had a mental illness, and who refused to participate in the study.

### Sample size estimation

The sample size for this study was obtained using a precision approach with a single proportion by using the following formula [21].

$$n=\frac{{z}^{2}p(100-p)}{{e}^{2}}$$

where, z = confidence interval (with 95% level of certainty) e = margin of error (5%), was reduced to increase the power of the study.

p = Prevalence of teenage pregnancy, P = 39% by UNFPA, [22] in Dodoma which was conducted to determine the prevalence of teenage pregnancies in Tanzania.

$${\text{Thus}}, n=\frac{{1.96}^{2}*39\left(100-39\right)}{{5}^{2}}\approx 451,$$

Whereby,

An addition of 20% for possible non-responses was made. Thus, a sample of 541 participants was expected in this study. However, 2 participants were excluded from the final analysis and, thus, the remaining sample was 539 participants who were included in the final analysis.

### Sampling procedure

A multi-stage sampling method was applied to randomly select the divisions, wards, villages, and finally households with adolescent girls. The primary sampling unit was districts followed by the division that was selected randomly by using the random numbers from the computer packages; the second cluster was wards that were selected at random from the list of each division. The third cluster was villages that were chosen randomly from the list in each ward. The last sampling unit was households with adolescent girls who were selected using a systematic sampling technique.

### Data collection methods

A structured questionnaire was used for data collection. The tool was constructed in the English language and translated into the Swahili language. The research assistants were trained for one day before the actual data collection process starts.

### Study variables and variables measurements

The dependent variable was teenage pregnancy.

A respondent was given one (1), if either confirmed pregnant at the time of data collection or has ever had become pregnant at the ages 13–19 years or Zero (0) if she was not pregnant during the time of data collection and has never had a pregnancy.

#### Independent variables

1. Socio-demographic variables (age, sex, marital status, education, occupation were considered) Parental education: the teenage girls were asked whether their parents (father/mother) attained any educational standard. It was measured as; no education, primary level of education, the secondary level of education, or post-secondary level of education. Parental occupation: the teenage girl was asked whether her father/mother is employed as; a farmer, business person, government/non-governmental organization, or other employment.
2. Behavioral factors: History of sexual and reproductive health, like age at first sexual intercourse, early marriage, and contraceptive use, perception of teenage pregnancy, family income, peer pressure, and casual sex assessed. Age at first sexual intercourse: the teenage girls were asked about their age in completed years at the first sexual encounter in life. Probes were used where she was not sure. Multiple sexual partners: this was determined by asking the teenager if she had a concurrent number of sexual partners at one time. Numbers were used to determine multiple sexual partners. The frequency of sexual intercourse: was determined by asking the teenage girl about the average number of sexual intercourse she has per week. Contraceptive use: was determined by asking whether the teenage girl regularly uses any contraceptive methods when having sexual intercourse.
3. Familial factors: Household socio-economic status: were determined by using proxy indicators where teenage girls were asked whether their household had possession of; a permanent building, electricity, solar power, vehicle, motorcycle, television, bicycle, radio, and animals. It was then categorized according to the value of the properties as high socioeconomic status, medium socioeconomic status, and low socioeconomic status. Marital status: the teenage girls were asked whether she was presently married or not. Parental divorce/separation: the teenage girl was asked whether her parents are divorced /separated. Domestic violence: the teenage girl was asked whether her parents, step-parents, siblings, or any adult living with her pushed, grabbed, kicked, or hit her with a fist, threatened to hurt her with a knife or other tools, slapped, or throw something at her most times. Any one of these responses was taken as domestic violence and no domestic violence if she did not mention anything. Physical neglect: the teenage girl was asked whether she gets enough food to eat at home, has torn clothes, has no sanitary pads when menstruating, has no money to buy breakfast and meals while at school when sick have no one to take her for treatment, parents/caretaker always drink too much alcohol and unable to cater for her basic needs, no one to protect and take care of her needs most times. Any one of these responses was taken as physical neglect and no physical neglect if nothing was mentioned.
4. Social factors: Peer pressure: the teenage girl was asked whether she has been forced/pressurized by friends to do what she didn’t want. It was measured as; never, often, quite often, and very often and further collapsed to no for never and yes for often/quite often/very often. Sexual abuse: the teenage girl was inquired about whether, a relative or an adult, had ever sexually touched her body, made her sexually touch their body, tried to have any sexual encounter with her, and forced her to have sexual intercourse. Any of these responses were taken as sexual abuse and no sexual abuse if any of these responses was not mentioned. Control over sexual intercourse: the teenage girl was asked whether she had equal say over sexual intercourse as compared to her partner as regards when to have sexual intercourse or not, and whether to use any contraceptive methods or not. The response was either a yes/no. Awareness of adolescent sexual and reproductive health: the teenage girl was asked whether her communities have been sensitizing on teenage sexual and reproductive health, and had information through reading newspapers, listening to the radio, or watching television. The response was either a yes/no. Perception of the cultural norm as regards sexual intercourse of teenage girls below 19 years: the teenage girl was asked whether her cultural norms allow sexual intercourse below the age of 19 years. The response was either a yes/no.
5. Knowledge was measured by summing up all correct questions among the eight questions used to assess knowledge of sexual and reproductive health; the percentage scores of more than 50% were regarded as having adequate knowledge otherwise had inadequate knowledge about sexual and reproductive health.

### Data analysis procedure

After data collection was entered into the Statistical Package for Social Science (SPSS Version 20.0). Descriptive statistics were employed to analyze and determine mean, frequency, and standard deviation. Frequency and percentage were employed to summarize categorical data (age group, level of education, types of contraceptives, level of occupation, parity category, and religious group). Knowledge of reproductive health was summarized using the figure. Factors associated with teenage pregnancy were established by using the Bivariate and Multivariate Logistic regression model, and a 95% confidence interval was used to describe the significance of associated factors. All variables which showed statistical significance in the bivariate model were subjected to the multivariate model to control possible confounding factors.

## Results

Enrolment and participation rate of the study participants.

**Fig. 1:**
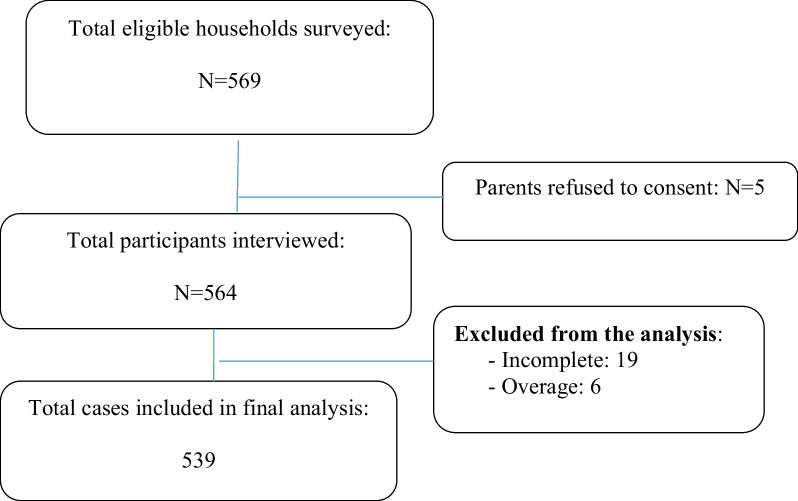
Enrolment of the study participants

A total of 569 households were eligible for this study. However, five (5) did not consent to the interview; this gave a participation rate of 99%. Twenty-five cases were excluded from the analysis (19 incomplete information, and 6 over the age > 19 years). Thus, five hundred thirty-nine (539) were included in the final analysis (Fig. 1).

### Socio-demographic characteristics

A total of 539 participants were analyzed in this study. The median age was 18 years with an interquartile range of 16–19 years old. The majority of 307 (57.0%) of the participants included in this study were from rural. The majority 357 (66.2%) were single, not married; 338 (62.7%) reached in primary school level; 337 (62.5%) were Christians. About 355 (65.9%) of the participants were entrepreneurs engaging in small businesses and minor activities for their income generation activities. About two-thirds, 412(76.4%) of the participants had both parents and 22(4.1%) were orphans. Regarding parents’ economic status, a high number of 411(76.3%) of the parents whose teens were included in this study had poor/underprivileged economic status (Table 1).

**Table 1:** Socio-demographic characteristics (N = 539)

Variables	Frequency	Percentage
Age (years)		
[Median; IQR]	[18; 16–19]	
13–14	29	5.4
15–17	204	37.8
18–19	306	56.8
Place of residence		
Urban	106	19.7
Semi-urban	126	23.4
Rural	307	57.0
Early marriage		
Single	357	66.2
Married	182	33.8
Highest level of education		
Non-formal	45	8.3
Primary level	338	62.7
Secondary level	156	28.9
Occupation		
Student	83	15.4
Peasant	73	13.5
Entrepreneur	355	65.9
Current employed	28	5.2
Ethnicity		
Gogo	170	31.5
Rangi	130	24.1
Others	239	44.3
Religion		
Christian	337	62.5
Muslim	202	37.5
Are parents alive		
Yes (both father and mother)	412	76.4
Yes, only father	19	3.5
Yes, only mother	86	16.0
No (orphan)	22	4.1
Mother’s education		
Non-formal	94	17.4
Primary	320	59.4
Secondary/above	125	23.2
Father’s education		
Non-formal	75	13.9
Primary	298	55.3
Secondary/above	153	28.4
Don't know	13	2.4
Parents economic status		
Adequate	128	23.7
Inadequate	411	76.3

### Knowledge of sexual and reproductive health among adolescents in the Dodoma region

**Fig. 2:**
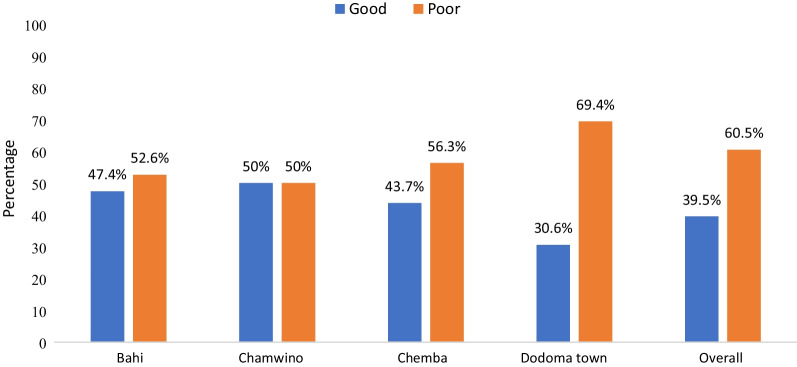
Level of knowledge on sexual reproductive health among adolescents in the Dodoma region

Overall knowledge of sexual and reproductive health was poor among adolescents in the Dodoma region. The results showed that the majority 326 (60.5%) had poor understanding/knowledge of sexual and reproductive health. Dodoma town had very low knowledge of sexual and reproductive health than any other district (Fig. 2).

### Adolescents' sexual and reproductive health practices in the Dodoma region

Most 507 (94.1%) already started sexual activities. Of five hundred seven who already engaged in sexual activities, 129 (25.4%) had very early sexual practices at the age before 14 years old, 219 (43.2%) at the age of 14–15 years; and only 159 (31.4%) started at the age above 15 years. 115 (21.3%) of the adolescents in this study reported having been practicing masturbation to reduce their sexual desire. Contraceptive use was very low among the adolescents enrolled in this study, only 120 (22.3%) reported currently using contraceptives, the main contraceptive used was a male condom 93 (17.3%) and oral pills 23 (4.3%); other contraceptives types were injectable 3(0.6%) and only one (0.2%) reported use Implanon (Table 2).

**Table 2:** Teenagers’ sexual and reproductive health practices in Dodoma region (N = 539)

Variables	N	%
Have you started or engaged in sexual activity?		
No	32	5.9
Yes	507	94.1
Early sexual practice (n = 507)		
13–15 years	348	68.6
> 15 years	159	31.4
Did you participate in the initiation rites?		
No	397	73.7
Yes	142	26.3
Early initiation rites (n = 142)		
< 13–15 years	98	18.2
> 15 years	44	8.1
Contraceptive use		
No	419	77.7
Yes	120	22.3
Current contraceptive use		
Male condom	93	17.3
Oral pills	23	4.3
Injectable	3	0.6
Implanon	1	0.2
None	419	77.7
Have you ever been engaging in masturbation to reduce your sexual desire?		
Yes	118	21.9
No	421	78.1

### The prevalence of teenage pregnancy in the Dodoma region

**Fig. 3:**
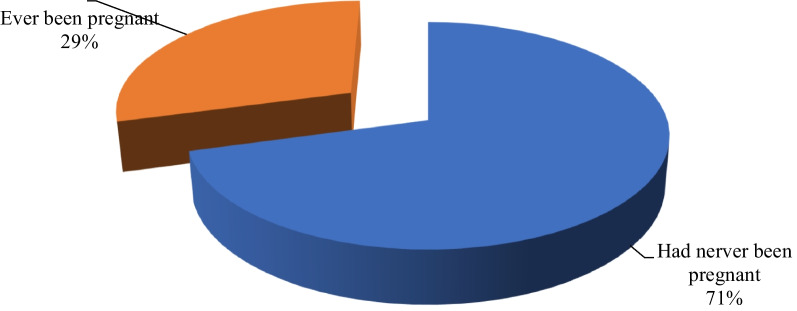
The prevalence of teenage pregnancy in the Dodoma region (N = 539)

The prevalence of teenage pregnancy was 158 (29%), which is equivalent to three in ten adolescents (Fig. 3).

### The relationship between socio-demographic characteristics and teenage pregnancy

From the chi-square test; socio-demographic factors that were associated with teenage pregnancy include age (p = 0.001), place of residence (p < 0.001), early marriage (p < 0.001), an education level (0.019); failure to obtain daily basic needs including clothes, food, and entertainment (p = 0.038); parents' economic status (p = 0.019); and the use of contraceptives (p < 0.001) (Table 3).

**Table 3:** The relationship between socio-demographic characteristics and teenage pregnancies (n = 539

Variables	N	Ever been pregnancy		$\chi^{2}$	P-value
		No	Yes		
Age (years)				13.776	0.001
13–14	29	29 (100.0)	0 (0.0)		
15–17	204	146 (71.6)	58 (28.4)		
18–19	306	206 (67.3)	100 (32.7)		
Place of residence				21.348	< 0.001
Urban	106	62 (58.5)	44 (41.5)		
Semi-urban	126	78 (61.9)	48 (38.1)		
Rural	307	241 (70.7)	66 (21.5)		
Early marriage				18.764	< 0.001
No	357	274 (76.8)	83 (23.2)		
Yes	182	107 (58.8)	75 (41.2)		
Level of education				7.932	0.019
Non formal	45	33 (73.3)	12 (26.7)		
Primary level	338	225 (66.6)	113 (33.4)		
Secondary level	156	123 (78.8)	33 (21.2)		
Ethnicity					
Gogo	170	124 (72.9)	46 (27.1)	3.000	0.223
Rangi	130	97 (74.6)	33 (25.4)		
Others	239	160 (66.9)	79 (33.1)		
Religion				3.660	0.056
Christian	337	248 (73.6)	89 (26.4)		
Muslim	202	133 (65.8)	69 (34.2)		
Mother’s education				28.663	< 0.001
Non-formal	94	64 (68.1)	30 (31.9)		
Primary	320	205 (64.1)	115 (35.9)		
Secondary/above	125	112 (89.6)	13 (10.4)		
Father’s education				25.858	< 0.001
Non-formal	75	36 (48.0)	39 (52.0)		
Primary	298	212 (71.1)	86 (28.9)		
Secondary/above	153	123 (80.4)	30 (19.6)		
Don't know	13	10 (76.9)	3 (23.1)		
Economic status				25.858	< 0.001
Good	128	104 (81.2)	24 (18.8)		
Poor	411	277 (67.4)	134 (32.6)		
Failure to obtain daily basic needs				4.301	0.038
No	131	102 (77.9)	29 (22.1)		
Yes	408	279 (68.4)	129 (31.6)		
Parents/caregivers’ economic status				5.533	0.019
Good	158	123 (77.8)	35 (22.2)		
Poor	381	258 (67.7)	123 (32.3)		

### The relationship between knowledge of sexual risk behavior and teenage pregnancy among adolescents

Variables of knowledge on sexual and reproductive health that showed a significant relationship with teenage pregnancy were awareness of whether a girl can get pregnant for the first time through sexual intercourse (p = 0.005), awareness of contraceptive use (p = 0.003), the perception that contraceptives influence one to get pregnancy (p < 0.001) and overall knowledge on sexual and reproductive health (p = 0.027) (Table 4).

**Table 4:** The relationship between knowledge about sexual risk behavior on teenage pregnancy (n = 539

Variables	N	Ever been pregnancy		$\chi^{2}$	P-value
		No	Yes		
An adolescent can get pregnant even if has not started menstruation				2.759	0.097
No	31	26 (83.9)	5 (16.1)		
Yes	508	355 (69.9)	153 (30.1)		
A girl can get pregnant at first time sexual intercourse				8.002	0.005
No	226	145 (64.2)	81 (35.8)		
Yes	313	236 (75.4)	77 (24.6)		
Aware of more sexual fertile period/dangerous period				1.064	0.302
No	316	218 (69.0)	98 (31.0)		
Yes	223	163 (73.1)	60 (26.9)		
Awareness of contraceptives use				8.911	0.003
No	79	67 (84.8)	12 (15.2)		
Yes	460	314 (68.3)	146 (31.7)		
Practices masturbation				1.184	0.277
Yes	115	86 (74.8)	29 (25.2)		
No	424	295 (69.6)	129 (30.4)		
Decision for sexual and reproductive health				0.002	0.962
Poor	284	201 (70.8)	83 (29.2)		
Good	255	180 (70.6)	75 (29.4)		
Ever concerned to conceive a pregnancy				0.409	0.522
No	150	103 (68.7)	47 (31.3)		
Yes	389	278 (71.5)	111 (28.5)		
Perceived that contraceptive influences one to get pregnancy				17.128	< 0.001
Yes	80	41 (51.2)	39 (48.8)		
No	459	340 (74.1)	119 (25.9)		
Contraceptive use				0.478	0.489
No	419	294 (70.2)	125 (29.8)		
Yes	120	87 (72.5)	33 (27.5)		
Knowledge of sexual and reproductive health				4.901	0.027
Adequate	213	162 (76.1)	51 (23.9)		
Inadequate	326	219 (67.2)	107 (32.8)		

### The relationship between cultural beliefs and teenage pregnancy among adolescence

Among cultural beliefs that were significantly associated with teenage pregnancy include the absence of restricted rules and regulations in the family (34% vs 25%; p = 0.032), early age of marriage practices (50% vs 27.7%; p = 0.004), and experienced sexual abuse (62.3% vs 13.5%, p < 0.001) (Table 5).

**Table 5:** Bivariate and multivariate logistic regression analysis on factors associated with teenage pregnancies (N = 539)

Variables	N	Teenage pregnancy	Crude OR(95%CI)	Adjusted OR(95%CI)	P-value
Place of residence					
Rural	433	114 (26.3)	1	1	
Urban	106	44 (41.5)	1.99 (1.27–3.10)	3.02 (1.60–5.68)	0.001
Mother’s education					
Primary/below	414	145 (35.0)	4.64 (2.48–8.68)	3.46 (1.47–8.11)	0.004
Secondary/above	125	13 (10.4)	1	1	
Father’s education (n = 526)					
Primary/below	373	125 (33.5)	2.07 (1.31–3.27)	1.32 (0.69–2.54)	0.402
Secondary/above	153	30 (19.6)	1	1	
Knowledge of sexual and reproductive health					
Good	213	51 (23.9)	1		
Poor	326	107 (32.8)	1.55 (1.05–2.30)	2.09 (1.2–3.63)	0.009
Family restricted rules against sexual practices					
Yes	238	81 (34.0)	1		
No	301	77 (25.6)	1.50 (1.03–2.18)	1.21 (.72–2.05)	0.470
Domestic violence					
Yes	153	43 (26.4)	1		
No	386	115 (35.0)	1.50 (1.02–2.21)	0.97 (0.58–1.63)	0.907
Failure to obtain daily basic needs including clothes, food, and entertainment					
No	131	29 (22.1)	1		
Yes	408	129 (31.6)	1.63 (1.02–2.59)	0.99 (0.50–1.99)	0.986
Parents’ economic status					
Good	158	35 (22.2)	1		
Poor	381	123 (32.3)	1.68 (1.08–2.59)	3.83 (1.77–8.30)	0.001
Peer group					
No	461	122 (26.5)	1		
Yes	78	36 (46.2)	2.08 (1.45–3.92)	2.92 (1.33–6.40)	0.007
Cultural influence including early marriage practices					
No	356	58 (20.6)	1		
Yes	183	100 (38.9)	2.46 (1.66–3.64)	2.58 (1.57–4.26)	< 0.0001
Experienced sexual abuse or enforced sexual practice					
No	282	58 (13.5)	1		
Yes	257	100 (62.3)	10.62 (6.46–17.44)	13.56 (7.98–23.06)	< 0.0001

### Factors associated with teenage pregnancy

From the adjusted odds ratio in multivariate analysis, factors that remained significantly associated with teenage pregnancy include urban residence [AOR: 3.02, 95%CI: (1.60–5.68), p = 0.001], low mothers’ education status of [AOR: 3.46, 95%CI: (1.47–8.11), p = 0.004]; Poor knowledge on sexual and reproductive health [AOR: 2.09, 95%CI: (1.2–3.63), p = 0.009]; Low economic status [AOR: 3.83, 95%CI: (1.77–8.30), p = 0.001]; Peer group [AOR: 2.92, 95%CI: (1.33–6.40), p = 0.007]; Early marriage [AOR: 2.58, 95%CI: (1.57–4.26), p < 0.0001]; and Sexual abuse [AOR: 13.56, 95%CI: (7.98–23.06), p < 0.0001], (Table 5).

## Discussion

The current study found the prevalence of teenage pregnancy at twenty-nine percent; this was low as compared Tanzania Demographic Health Survey (TDHS) in 2016, which reported a high prevalence of teenage pregnancy in the Dodoma region that was thirty-nine percent [22], the current finding was ten percent lower than the previous report by TDHS. This difference can be explained by the methodological approaches used in these two pieces of information, the DHS report included a larger sample size and it was done over five years before the current study On another hand, the current findings reported a very high prevalence of teenage pregnancy than the global estimate, World Health Organization indicates a global prevalence of teenage pregnancy 6.5% for all adolescents aged 15–19 years; however more than 90% of these occur in LMICs [23]. Similarly, in the study done in East Africa in Kenya, Uganda, and Tanzania by Sarah et al. [24] reported a high rate of women giving birth before 20 years were 47%, 57%, and 56%, respectively. This is supported by the World Health Organization report which shows that about 90% of teen pregnancy occurs in LMICs, and most are unintended pregnancies [23].

The majority of teenagers reported had ever heard information about reproductive health however, about two-fifths were only found to have good knowledge of sexual reproductive health while the majority had limited knowledge and were not even able to indicate the risk of pregnancy period or dangerous period for fertilization, do not aware about unsafe sexual practices including unprotected sexual intercourse and less use of contraceptives. The same agreement with the study in Saudi Arabia, which revealed that 54% of teenagers had less than 15 years of and 70.7% of those greater than 15 years had poor sexual health knowledge [25]. This is corresponding to numerous studies in Low and Middle-Income Countries (LMICs) particularly in Africa [3, 26]. Low knowledge can be contributed to inadequate policy and poor implementation of reproductive health services including reproductive health education and counseling among teenagers such as education and counseling about sexuality and safe sexual practices. The current study reported that major sources of information about reproductive health include radio or television, social media (such as Facebook, WhatsApp), and friends/relatives; a minority indicated other sources like schoolteachers, posters or leaflets, and health care providers. The source of information on reproductive health is still insufficient to provide adequate knowledge on sexual and reproductive health, only a minority reported having information from health care providers and this indicates that only a minority of teens can meet health care providers for health service.

Early sexuality is common among teens, the current study found that more than eighty percent of teens were already engaged in sexual practices, and about twenty-five percent reported starting sexual practices at a very early age before 14 years old. Similarly, the study by AlQuaiz, Kazi and Muneef [10], in Riyadh-Saudi Arabia revealed that poor knowledge of reproductive health and practices was the main contributor to teen pregnancy. In addition to that, the study by Darroch et al. found that most unplanned pregnancies experienced by teenage girls arise among those who are using no birth control measures [27]. This is comparable with the current study, which showed that contraceptive use was very low among the adolescents enrolled in this study, whereas 22.3% had been using contraceptives in their lifetimes with the main contraceptive type being male condoms and oral pills. The major reasons for not using contraceptives included fear of being known to people that they use contraceptives, fear from parents, and fear of being called a prostitute. This indicates that the majority of teens lack confidence in contraceptive use and calls for an urgent need to provide more education about contraceptive use among teens from the study site or in Tanzania at large, particularly in schools or having mass education in the community for teens.

Low mothers’ education was found significant predictor of teenage pregnancy. Low education of parents (mothers) is strongly linked with less education to teenagers and therefore puts them at risk of early pregnancy and early motherhood. This is consistent with the study by Mohr et al. [28] who reported that less-educated teens increased the risk of getting pregnant than those going to an advanced level or high level. Another study by Khoza linked education and knowledge on sexual and reproductive health and indicated that girls who are less educated have inadequate knowledge about the dangers of engaging in sexual activities at an early age and also they don’t know how to take care of their bodies as well as issues concerning family planning methods thus end-up getting pregnant [29].

Similarly, the current study showed that poor knowledge of sexual and reproductive health was a strong predictor of teenage pregnancy. Another study in Sri Lanka also showed that low levels of knowledge of sexual and reproductive health among adolescents aged 16–19 added to the high rate of teenage pregnancies [30]. Correspondingly numerous studies in developing countries by [6, 28] showed that there is a direct association between knowledge of reproductive health and teenage pregnancy*.*

The current study strongly indicated cultural practices such as including early marriage practices were the significant predictor for teenage pregnancy after adjusting other confounders such as age, education residence etc. This is in agreement with the study conducted in Nepal and Iran which showed that many young people particularly unmarried girls are affected by social and cultural norms that impose barriers towards the risk of sexuality hence increasing the risk of early pregnancy and early motherhood [31, 32]. The issue of cultural influence is a direct factor, for example, families in rural areas and with low education and economic status would prefer to force their young girls towards early marriage for marriage pride and do not consider the next risk of their teens [33].

This study found a significant influence of parents' economic status on teenage pregnancy, whereby teenagers whose parents were poor had a significant risk of early pregnancy than those whose parents had good economic status or wealth. This is in agreement with the study by [34] who stated that family income inequalities tend to create early sexual activity involvement among teens and therefore increase the risk of early motherhood. Another study [35] in Sri Lanka also reported similar findings, through which it was observed that families with a low income had a significant risk of early sexual activity involvement and teen pregnancy [34]. This can be explained due to the following reasons, low income is linked with failure to obtain daily basic needs including clothes, food, and entertainment which make it creates an environment for dropping from their studies, some run away from homes looking for jobs (house girls), increase risk of raping and some get easy to accept men hoping for support that all do creates an environment for early sexual practices and pregnancy. This is also supported by the previous findings [34, 36].

Corresponding to the findings from the previous studies by [11, 37] showed that, families at the poverty level fail to offer basic needs to their children or teens increases the risk of pre-marital sex and therefore increases more risk of early sexual activity [11, 37]. Another study by [32] reported that the majority of teenage girls in families with poor economic background find it difficult to fulfill their expectations and this poses the risk of early involvement in sexual activity which not cause only early pregnancy but also increase the risk of sexually transmitted infections such as HIV/AIDS, risk of abortions, and increased risk of life-threatening maternal complications and mortality [32].

Another risk factor was peer group, the teenagers who reported having been engaging in peer groups such as social activities like disco, going to schools together, sports, and going to similar works together with their peers (boys and girls) showed statistically significant contribution towards teenage pregnancy. Similar to the study by Neal et al. [38] which indicated that peer pressure plays a big role in initiating sexual activity, which frequently ends in early pregnancies, peer pressure provides the chances of having frequent sexual actions increases, forced sex initiation that directly put girls at risk of teenage pregnancy [34, 37]. This is corresponding to the current findings, which have shown that sexual abuse such as rape or forced sexual practices was also strongly associated with early sexual practices and teenage pregnancy.

The study was not without limitations. Being a cross-sectional study, it failed to have a causal effect relationship. The authors are recommending an interventional study to come up with a cost-effective strategy to address the challenge of teenage pregnancy in Dodoma. Also, another limitation of the study was the recall bias, respondents could have underreported or overreported because the study depended on self-reported information from past events. This was minimized by having.

## Conclusion

This study found a high prevalence of teenage pregnancy with limited knowledge and practices of sexual and reproductive health. Predictors for teenage pregnancy from the study site were urban residence, parents' poor economic status/poverty, low parent’s education status, low knowledge of sexual and reproductive health, peer group, cultural influence including early marriage practices, and Sexual abuse. An innovative intervention study to come up with an effective strategy to address the challenge of teenage pregnancy in Dodoma is highly recommended.

## Data Availability

The data set is available and it can be produced upon request.

## Abbreviations

AIDS: Acquired Immunodeficiency Syndrome
AOR: Adjusted Odds Ratio
DHS: Demographic and Health Survey
HIV: Human immunodeficiency virus
LMIC: Low and middle income countries
RAS: Regional Administrative Secretary
SPSS: Statistical Package For The Social Sciences
SSA: Sub-Saharan Africa
TDHS: Tanzania Demographic and Health Survey
UNICEF: United Nations International Children's Emergency Fund
WHO: World Health Organization
